# Parent-Child Resemblance in Weight Status and Its Correlates in the United States

**DOI:** 10.1371/journal.pone.0065361

**Published:** 2013-06-10

**Authors:** Yinghui Liu, Hsin-jen Chen, Lan Liang, Youfa Wang

**Affiliations:** 1 Johns Hopkins Global Center on Childhood Obesity and Center for Human Nutrition, Department of International Health, Johns Hopkins Bloomberg School of Public Health, Baltimore, Maryland, United States of America; 2 Center for Financing, Access and Cost Trends, Agency for Healthcare Research and Quality, Rockville, Maryland, United States of America; University of Missouri-Kansas City, United States of America

## Abstract

**Background:**

Few studies have examined parent-child resemblance in body weight statu**s** using nationally representative data for the US.

**Design:**

We analyzed Body Mass Index (BMI), weight status, and related correlates for 4,846 boys, 4,725 girls, and their parents based on US nationally representative data from the 2006 and 2007 Medical Expenditure Panel Survey (MEPS). Pearson partial correlation coefficients, percent agreement, weighted kappa coefficients, and binary and multinomial logistic regression were used to examine parent-child resemblance, adjusted for complex sampling design.

**Results:**

Pearson partial correlation coefficients between parent and child’s BMI measures were 0.15 for father-son pairs, 0.17 for father-daughter pairs, 0.20 for mother-son pairs, and 0.23 for mother-daughter pairs. The weighted kappa coefficients between BMI quintiles of parent and child ranged from −0.02 to 0.25. Odds ratio analyses found children were 2.1 (95% confidence interval (CI): 1.6, 2.8) times more likely to be obese if only their father was obese, 1.9 (95% CI: 1.5, 2.4) times more likely if only their mother was obese, and 3.2 (95% CI: 2.5, 4.2) times more likely if both parents were obese.

**Conclusions:**

Parent-child resemblance in BMI appears weak and may vary across parent-child dyad types in the US population. However, parental obesity status is associated with children’s obesity status. Use of different measures of parent-child resemblance in body weight status can lead to different conclusions.

## Introduction

Approximately one-third of American children are obese or overweight, making childhood obesity prevention a public health priority [1], [2], [3]. Numerous studies have explored genetic and environmental contributions to childhood obesity. However, complete understanding of the complex biological, social, environmental, and behavioral causes of childhood obesity remains elusive [4].

Family is an important target of childhood obesity interventions. Both shared genetic backgrounds and shared environmental factors can result in children and parents being alike in weight status [4]. Furthermore, family members and, in particular, parents exert important, direct influences on children’s dietary intakes [5], [6], [7] and physical activity levels [8], which thereby affect their child’s energy balance and weight status. To explore the familial factors that contribute to childhood obesity, studies have examined parent-child resemblance in body mass index (BMI) and obesity status [9], [10], [11], [12], [13], [14]. However, these studies have observed mixed results. Some studies report strong parent-child associations [15], [16] and others report weak or no such relationships [13]. Among five studies that examined parent-offspring resemblance in long-term changes of BMI or obesity status, four found a correlation between BMI changes [9], [15], [17], [18], while one did not [19]. Previous research examining racial/ethnic differences in parent-child BMI resemblance in the US has also yielded mixed results. A study in 1983 observed higher parent-offspring correlations in BMI for whites (r = 0.23–0.29) than blacks (r = −0.07) [20], but a later study reported the opposite (r = 0.15 for whites and 0.18 for blacks, p<0.05) [11].

Previous studies testing parent-child resemblance in weight status are predominantly based on specific populations and/or composed of small sample sizes; thus, their results are not generalizable. To our knowledge, there have been only two studies (one in the US [21] and the other in Finland [18]) to date that have examined parent-child resemblance in body weight status using nationally representative data. However, neither study further examined possible factors with potential effects on the extent of the resemblance.

This study examined parent-child resemblance in BMI and body weight status using nationally representative data for the US, and investigated potential sources of variation across the population (e.g., race/ethnicity, age, sex, and socioeconomic status [SES]). We hypothesized resemblance would differ between socio-demographic and socioeconomic groups.

## Methods

### Data Source

The Medical Expenditure Panel Survey (MEPS) is a nationally representative survey concerning medical care costs and expenses across the United States. A new sample of households is selected each year from participating households in the preceding year’s National Health Interview Survey (NHIS) pool and studied over a two-year time frame. The data from two consecutive MEPS samples are combined to produce a consolidated annual dataset for each calendar year. Because of their origin, MEPS data inherit the complex sampling design of the NHIS. Greater detail regarding the MEPS study design and its data collection procedures have been documented elsewhere [22]. The MEPS Household Component (HC) includes data on participants’ demographic characteristics, health conditions, health status, income, employment, and medical care use and expenditures. Since 2000, the MEPS has also collected self-reported body weight and height data. For the present study, we used the MEPS HC annual data files for 2006 and 2007.

### Study Population

We included children aged 6–17 years and their parents with complete BMI and other key data. Subjects were excluded if: 1) BMI was missing; 2) BMI was extreme (child BMI was <7.5 kg/m^2^ or >43.1 kg/m^2^; adult BMI was <13.0 kg/m^2^ or >54.3 kg/m^2^); 3) subject was pregnant; 4) subject had a diagnosis of a condition or disease such as cancer, AIDS, or thyroid disease with the potential to affect BMI; or 5) subject was >65 years of age. The final sample used for analysis included 4,846 boys, 4,725 girls, and their parents.

### Body Weight Status Measures

BMI was calculated as weight (kg)/[height (m)]^2^. An adult household representative reported height and weight for all household members, including children. Child’s body weight status was classified using age- and sex-specific BMI percentiles using the 2000 US Centers for Disease Control and Prevention (CDC) Growth Charts [23]. A child was classified as overweight if his/her BMI-for-age percentile was ≥85^th^ percentile and <95^th^ percentile and obese if his/her BMI was ≥95^th^ percentile. BMI-for-age Z-scores were also calculated for each child based on the 2000 CDC Growth Charts. BMI-for-age z-scores indicate how many standard deviations one’s BMI is from the population mean BMI for the same age and sex. For adult/parent’s body weight status, overweight was classified as having a BMI ≥25 kg/m^2^ and <30 kg/m^2^ while obese was classified as having a BMI ≥30 kg/m^2^.

### Explanatory Variables

Parental body weight status and BMI were the major explanatory variables used to examine parent-child weight resemblance. Other covariates included: 1) child’s age (categorized into 3 groups: 6–9 years, 10–14 years, and 15–17 years); 2) annual household income (categorized into 3 groups and defined by the household income relative to the percentage of the federal poverty line [FPL] applicable according to family size and composition: low- [<200% FPL], middle- [between 200%–400% FPL] and high-income [≥400% FPL]); and 3) parental socio-demographic characteristics, including a) race/ethnicity (non-Hispanic [NH] white, NH black, Hispanic, and other), b) education level (≤11, 12, 13–15, and ≥16 years), c) age (20–34, 35–49, and 50–64 years), and d) marital status (married or other). Based on the answers (yes/no) to the question “Whether or not [person] currently spends half an hour or more in moderate to vigorous physical activity (PA) at least three times a week?”, parents were grouped as <3 times physically active or ≥3 times physically active per week.

### Statistical Analysis

We applied several statistical techniques to study parent-child resemblance in BMI and weight status. All analyses were conducted in SAS release 9.2 or Stata version 10.0, using survey commands to take into account the complex sampling design effect and survey weights so as to produce nationally representative estimates [24]. For all tests, we considered p<0.05 statistically significant.

First, we estimated the adjusted Pearson correlation coefficients between the standard score of children BMI-for-age Z-score and the standard score of parent BMI, stratified by child and parent characteristics. These coefficients were obtained from linear models regressing the standard score of child’s BMI-for-age Z-score on the standard score of parent’s BMI, controlling for child’s age, annual household income (%FPL), and parent’s age, education level, race/ethnicity, marital status, and/or physical activity. When a variable was used as the stratifying characteristic, it was not included as a covariate. We tested for between-group differences in Pearson correlation coefficients based on interaction terms composed of parent BMI standard score and the stratifying variable of interest. To explore the possibility of age trends, we also fit locally weighted least squares (LOWESS) curves of Pearson correlation coefficients against child age for each parent-child dyad.

Second, we calculated the percent agreements and weighted kappa coefficients between the age- and sex-specific quintiles of the child’s BMI-for-age Z-score and sex-specific quintiles of the parent’s BMI. These statistics measure the level of agreement between two measurements beyond what would be expected by chance. Observed percent agreement (%) is the percentage of pairs in the diagonal, concordant cells of a two-way 5×5 quintile table over the total number of pairs in the table. In a 5×5 table, if there is no resemblance between parent and child BMI, the sample would equally distribute in the cells, resulting in an expected percent agreement 5/25 or 20%.

Weighted kappa (κ) coefficients test the difference between observed and expected percent agreement, with application of the Cicchetti-Allison weight matrix (where “1” is the weight for the cells in the diagonal, and 0.75, 0.5, 0.25 and 0 are the values, respectively, for the cells moving sequentially away from the diagonal). Weighted kappa values can be interpreted as follows: κ<0.20 =  poor agreement, κ between 0.20–0.40 =  fair agreement, κ between 0.40–0.60 =  moderate agreement, κ between 0.60–0.80 =  good agreement, and κ≥0.80 =  very good agreement [25], [26]. Survey sampling weights were applied to obtain population-representative estimates for the percent agreements and weighted kappa coefficients. We used Fay’s balanced repeated replication (BRR) method to estimate appropriate variances for these non-parametric statistics; Fay’s coefficient was set to 0.5 [27].

Third, to assess how parental obesity status might predict children’s obesity status, we applied logistic regression models for 5,900 children from dual-parent families. The dependent variable was the child’s obesity status. The primary independent variable was parental obesity status (only father was obese, only mother was obese, both father and mother were obese, and neither was obese [reference group]). The models were adjusted for child and parental socio-demographic and socioeconomic variables. The analyses were also stratified by the child’s age, household income, and parental characteristics.

Finally, we created a nominal outcome variable based on the pattern of concordance between child and parent’s body weight status. Four groups were created and defined as follows: (1) the reference group, in which neither the parent (father or mother) nor the child was overweight (where BMI≥25 kg/m^2^ for adults and BMI-for-age percentile ≥85^th^ for children), (2) a group in which only the parent (father or mother) and not the child was overweight, (3) a group in which only the child and not the parent was overweight, and (4) a group in which both the parent (father or mother) and the child were both overweight. We analyzed father-child and mother-child dyads separately. We used Stata 10.0 to fit multinomial logistic regression models to explore potential factors that might predict the pattern of concordance.

## Results

Boys were more likely to be obese (20.6% vs. 14.8%) than girls were. More than 30% of the parents in the sample had BMIs that classified them as obese. Mothers in the mother-son and mother-daughter pairs had similar BMIs. Compared to the fathers in the father-daughter dyads, those in the father-son dyads had higher BMIs and were less likely to be married. No significant differences were observed between mothers in the mother-son and mother-daughter dyads or between fathers in the father-son and father-daughter dyads by age, education level, race/ethnicity, or frequency of moderate-to-vigorous physical activity (Table 1).

**Table 1:** Characteristics of US children and their parents.

Characteristics^1^	Boys	Girls	*p-value* 2
**1. Child characteristics**	n = 4846	n = 4725	
Child age (years)	12.0 (11.8, 12.1)	12.0 (11.8, 12.2)	0.903
6–9	28.0 (26.1, 29.9)	26.8(24.9, 28.7)	0.263
10–14	42.5 (40.5, 44.4)	44.7(42.8, 46.7)	
15–17	29.5 (27.8, 31.2)	28.4(26.6, 30.3)	
Child BMI (kg/m^2^)	20.7 (20.5, 20.9)	20.3(20.1, 20.5)	0.002
Prevalence of overweight and obesity, based on 2000 CDC Growth Charts			
<85^th^ percentile (%-ile)	63.2 (61.3, 65.1)	70.3(68.5, 72.2)	<0.0001
Between 85^th^≤BMI<95^th^ %-ile	16.2 (14.8,17.6)	14.9(13.6, 16.2)	
BMI ≥95^th^%-ile	20.6(19.1, 22.1)	14.8(13.5, 16.1)	
Annual household income, %			
Low income	32.3 (29.1, 34.7)	33.2(30.9, 35.5)	0.399
Middle income	34.9(32.8, 37.1)	35.7(33.5, 38.0)	
High income	32.8(30.1, 35.4)	31.0 (28.5, 33.6)	
**2. Characteristics of children's mothers**	n = 4585	n = 4522	
Age (years)	40.1(39.8, 40.5)	40.0 (39.7, 40.3)	0.558
20–34	20.7 (18.9, 22.5)	21.1(19.0, 23.1)	0.921
35–49	71.2 (69.2, 73.2)	71.1(68.6, 73.5)	
50–65	8.1 (6.8, 9.3)	7.8(6.6, 9.1)	
BMI (kg/m^2^)	27.4(27.1, 27.8)	27.5(27.2, 27.8)	0.761
Prevalence of weight status			
Underweight, BMI <18.5	2.0(1.3, 2.7)	2.3(1.5, 3.0)	0.64
Normal weight, 18.5< BMI ≤25	40.0(37.4, 42.6)	38.8(36.3, 41.3)	
Overweight, 25.0≤BMI <30	27.3(25.2, 29.5)	28.7(26.4, 31.0)	
Obese, BMI ≥30.0	30.6(28.3, 33.0)	30.2(27.9, 32.5)	
Education level, %			
≤11 years (high school)	15.2(13.5, 17.0)	15.4(13.4, 17.3)	0.997
12 years (high school)	29.2(26.8, 31.7)	29.0 (26.4, 31.5)	
13–15 years (some college)	26.7(24.2, 29.2)	26.7(24.3, 29.1)	
≥16 years (≥college)	28.8(26.0, 31.6)	28.9(26.3, 31.6)	
Race/ethnicity, %			
Non-Hispanic (NH) white	63.8(60.9, 66.7)	62.9(60.0, 65.8)	0.389
NH black	13.2(11.5, 15.0)	14.7(13.0, 16.5)	
Hispanic	17.7 (15.7, 19.7)	17(14.8, 19.1)	
Other	5.3(3.9, 6.6)	5.4(4.0, 6.7)	
Marital status, %			
Married	74.7(72.4, 76.9)	73.2(70.8, 75.7)	0.283
Other	25.3(23.1, 27.6)	26.8(24.3, 29.2)	
Frequency of moderate to vigorous physical activity per week, %			
<3 times	42.9(40.5, 45.3)	44.6(42.3, 46.9)	0.225
≥3 times	57.1(54.7, 59.5)	55.4(53.1, 57.7)	
**3. Characteristics of children's fathers**	n = 3406	n = 3229	
Age (years)	42.8(42.4,43.2)	42.5(42.1, 42.9)	0.181
20–34	13.1(11.3, 14.8)	12.8(10.7,14.8)	0.625
35–49	69.5(67.0, 71.9)	70.9(68.1, 73.7)	
50–65	17.5(15.2, 19.8)	16.3(14.3, 18.3)	
BMI (kg/m^2^)	28.6(28.3,28.9)	28.3(28.0, 28.6)	0.039
Prevalence of weight status			
Underweight, BMI <18.5	0.3 (0.0, 0.7)	0.3(0.0, 0.7)	0.236
Normal weight, 18.5< BMI ≤25	21.0 (18.7, 23.3)	23.9(21.4, 26.4)	
Overweight, 25.0≤BMI <30	45.6(42.9, 48.4)	44.8(42.1, 47.6)	
Obese, BMI ≥30.0	33.0 (30.0, 36.0)	31.0 (28.0, 33.9)	
Education level,%			0.208
≤11 years (high school)	15.0 (13.0, 17.1)	14.3(12.2, 16.5)	
12 years (high school)	31.1(28.3, 33.9)	31.4(28.6, 34.3)	
13–15 years (some college)	23.6(20.7, 26.4)	21.2(18.7, 23.7)	
≥16 years (≥college)	30.3(26.9, 33.6)	33.0 (29.9, 36.2)	
Race/ethnicity, %			0.213
Non-Hispanic (NH) white	68.9(66.0, 71.9)	69.1(66.1, 72.1)	
NH black	9.1(7.6, 10.6)	10.2(8.6, 11.7)	
Hispanic	16.5(14.4, 18.5)	14.8(12.6, 16.9)	
Other	5.5(4.1, 6.8)	6.0 (4.5, 7.4)	
Marital status, %			0.006
Married	90.7(89.0, 92.5)	93.3(92.0, 94.5)	
Other	9.3(7.5, 11.0)	6.7(5.5, 8.0)	
Frequency of moderate to vigorous physical activity per week, %			
<3 times	37.6(34.6, 40.5)	37.8(34.9, 40.7)	0.89
≥3 times	62.4(59.5, 65.4)	62.2(59.3, 65.1)	?

1 Statistics are reported either as means with 95% Confidence Interval (CI) for continuous variables or percentages with their 95% Confidence Interval (CI) for categorical variables.

2 Chi-square tests were used to test for between-group differences for categorical variables; t-tests were used for continuous variables.

Source: Medical Expenditure Panel Survey, 2006–2007.

### Correlation between Parent and Child BMI

**Figure 1:**
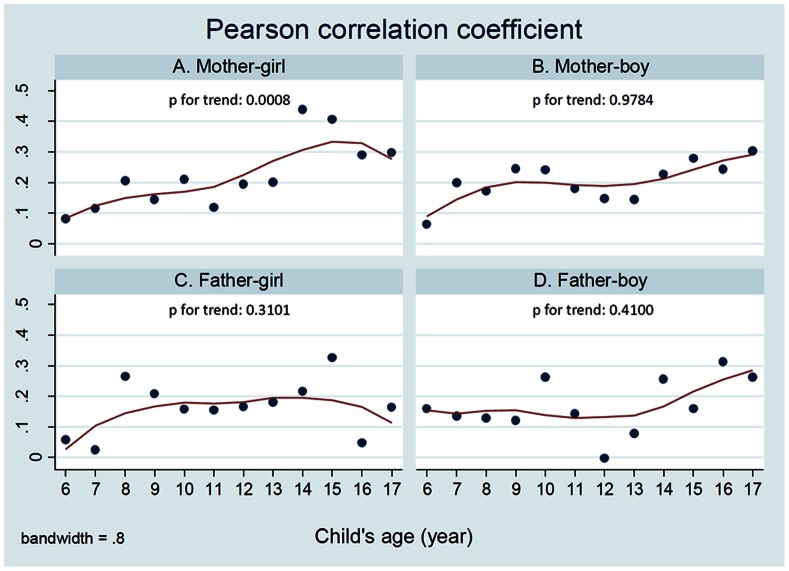
Age trends in the Pearson partial correlation coefficient between parent’s BMI and child’s BMI for age by dyad types. X axis: Kid’ age. Y axis: Correlation coefficient r. P value for dyads: P  = 0.0008 for mother-daughter pairs; P  = 0.9784 for mother-son pairs; P  = 0.3101 for father-daughter pairs; P  = 0.4100 for father- son pairs.

As shown in Table 2, the overall Pearson partial correlation coefficients (r) between BMI in the mother-child dyads were larger than those in the father-child dyads. Correlation coefficients ranged from 0.15 in the father-son dyads to 0.23 in the mother-daughter dyads. The coefficients also varied by other characteristics. For example, greater correlations were found in father-son pairs in which the father had a low educational level (≤11 years) (r = 0.28) or was married (r = 0.16). Higher correlations were also observed in father-daughter dyads in which the father was between 35–49 years old (r = 0.20). In the mother-child dyads, correlations were greater in mother-son dyads in which maternal educational level was low (r = 0.22 for ≤11 years of education, r = 0.23 for 12 years, and r = 0.23 for 13–15 years), as well as in mother-daughter dyads in which the child was in the 15–17 year-old group (r = 0.30). Figure 1 shows that the partial correlation coefficients between mother and daughter BMI increased with daughter’s age, but similarly significant age trends were not seen in the other dyad types.

**Table 2:** Pearson partial correlation coefficients (r)^1^ between parent and child BMI measures by dyad type and various socio-demographic and socioeconomic characteristics.

Characteristics	Father-son		Father-daughter		Mother-son		Mother-daughter	
	n	r (SEM)^2^	n	r (SEM)	n	r (SEM)	n	r (SEM)
Total	3329	0.15 (0.03)	3161	0.17 (0.02)	4475	0.20 (0.02)	4424	0.23 (0.02)
Child age (years)								
6–9	974	0.14 (0.05)	902	0.14 (0.04)	1289	0.19 (0.04)	1222	0.16 (0.04)^3^
10–14	1466	0.14 (0.03)	1380	0.18 (0.04)	1973	0.19 (0.03)	1934	0.22 (0.03)
15–17	889	0.18 (0.04)	879	0.17 (0.04)	1213	0.23 (0.03)	1268	0.30 (0.03)^ref^
Parental age (years)								
20–34	567	0.07 (0.05)	526	0.06 (0.05)^4^	1201	0.21 (0.03)	1192	0.19 (0.04)
35–49	2205	0.18 (0.04)	2156	0.20 (0.03)^ref^	2943	0.20 (0.03)	2925	0.23 (0.03)
50–65	557	0.16 (0.05)	479	0.13 (0.04)	331	0.21 (0.07)	307	0.28 (0.07)
Parental race/ethnicity								
NH white	1694	0.12 (0.03)	1609	0.16 (0.03)	2020	0.18 (0.03)	1949	0.24 (0.03)
NH black	417	0.15 (0.07)	427	0.20 (0.07)	821	0.24 (0.05)	881	0.21 (0.05)
Hispanic	1018	0.30 (0.07)	950	0.20 (0.05)	1403	0.25 (0.06)	1393	0.19 (0.04)
Other	200	0.27 (0.12)	175	0.20 (0.09)	231	0.25 (0.08)	201	0.21 (0.09)
Parental education year								
≤11 years (high school)	804	0.28 (0.08)^5^	761	0.14 (0.07)	1125	0.22 (0.04)	1151	0.23 (0.04)
12 years (high school)	1007	0.09 (0.04)^ref^	969	0.19 (0.04)	1333	0.23 (0.04)^ref^	1279	0.22 (0.04)
13–15 years (some college)	675	0.09 (0.04)	600	0.18 (0.05)	1062	0.23 (0.04)	1025	0.25 (0.04)
≥16 years (≥college)	789	0.19 (0.04)	790	0.15 (0.04)	908	0.11 (0.04)^6^	931	0.22 (0.05)
Household income								
Low income	1292	0.13 (0.04)	1235	0.14 (0.03)	2240	0.22 (0.04)	2207	0.24 (0.03)
Middle income	1119	0.17 (0.04)	1090	0.19 (0.04)	1293	0.21 (0.03)	1334	0.24 (0.03)
High income	918	0.15 (0.03)	836	0.17 (0.04)	942	0.14 (0.04)	883	0.19 (0.04)
Parental marital status								
Other	281	0.03 (0.06)^7^	254	0.25 (0.08)	1386	0.22 (0.04)	1469	0.27 (0.04)
Married	3048	0.16 (0.03)^ref^	2907	0.16 (0.02)	3089	0.19 (0.03)	2955	0.21 (0.03)
Parental MVPA frequency/week								
<3 times	1987	0.14 (0.04)	1872	0.15 (0.03)	2374	0.21 (0.03)	2278	0.20 (0.03)
≥3 times	1332	0.18 (0.04)	1279	0.19 (0.03)	2087	0.19 (0.03)	2134	0.25 (0.03)

1 The r was adjusted for child's age and parent's age, ethnicity, education, household income, marital status, and physical activity. In the stratified analysis, partial correlation coefficients were calculated adjusting for the same variables except the stratification variable. For example, for the father-son correlation, we controlled for child age and father's characteristics as described above. For the father-son correlation stratified by household income, we controlled child age and father's characteristics with exception of household income.

2 SEM: standard error of measurement.

3–7 Significantly different from the reference group (p<0.05), based on the interaction terms of parent BMI standard score and the strata of interest.

NH: non-Hispanic; ref: reference group.

Source: Medical Expenditure Panel Survey, 2006–2007.

### Agreement between Parent’s BMI Quintile and Child’s BMI-for-age Z-score Quintile

Table 3 shows percent agreements and weighted kappa coefficients between parent and child quintiles varied by parent-child dyad characteristics. Percent agreement was the lowest (at 21.6%) for the father-son dyads, 22.5% for the father-daughter pairs and 23.7% for the mother-son pairs, and 25.5% for the mother-daughter pairs. Weighted kappa coefficients (κ) were higher (i.e. 0.18) for the mother-daughter dyads compared to other dyads: the weighted kappa coefficient was 0.08 for the father-son pairs, 0.10 for the father-daughter pairs, and 0.12 for the mother-son pairs. Fair levels of agreement (κ between 0.20–0.40) were observed in father-son pairs in which the father’s education was ≤11 years (κ = 0.22), in NH black father-daughter pairs (κ = 0.25), in NH black mother-daughter pairs (κ = 0.24), in mother-daughter pairs with low household income (κ = 0.21), and in mother-daughter pairs in which the mother engaged in more frequent MVPA (κ = 0.20). Agreement was poor (κ<0.20) for all other cases. There was no agreement in mother-son pairs where the mother was aged ≥50 years old (κ = −0.02).

**Table 3:** Percent agreement (%) and weighted kappa coefficients between parent and children BMI quintiles by dyad type and characteristics^1^.

Characteristics	Father-son		Father-daughter		Mother-son		Mother-daughter	
	Percent Agreement	Weighted kappa coefficient	Percent Agreement	Weighted kappa coefficient	Percent Agreement	Weighted kappa coefficient	Percent Agreement	Weighted kappa coefficient
	%^2^ (SEM )^3^	weighted *K* 4 (SEM )	% (SEM )	weighted *K* (SEM )	% (SEM )	weighted *K* (SEM )	% (SEM )	weighted K(SEM)
Total	21.6 (0.1)	0.08 (0.00)	22.5 (0.1)	0.10 (0.00)	23.7 (0.1)	0.12 (0.00)	25.5 (0.1)	0.18 (0.00)
Child age (years)								
6–9	18.9 (0.2)	0.12 (0.02)	25.8 (0.3)	0.01 (0.02)	25.6 (0.2)	0.15 (0.01)	26.3 (0.3)	0.13 (0.01)
10–14	23.2 (0.2)	0.15 (0.02)	22.6 (0.1)	0.16 (0.01)	25.2 (0.2)	0.14 (0.00)	26.4 (0.1)	0.19 (0.01)
15–17	22.0 (0.2)	0.14 (0.02)	19.0 (0.3)	0.08 (0.01)	19.9 (0.2)	0.11 (0.01)	23.2 (0.1)	0.17 (0.01)
Parental age (years)								
20–34	17.3 (0.2)	0.08 (0.01)	26.1 (0.5)	0.08 (0.02)	26.8 (0.2)	0.15 (0.01)	23.6 (0.2)	0.12 (0.01)
35–49	22.0 (0.1)	0.14 (0.02)	22.5 (0.1)	0.12 (0.00)	23.5 (0.1)	0.16 (0.01)	26.2 (0.1)	0.18 (0.00)
50–65	23.5 (0.4)	0.15 (0.01)	19.9 (0.5)	0.02 (0.02)	17.7 (0.6)	−0.02 (0.02)	23.5 (0.8)	0.16 (0.03)
Parental education (years)								
≤11	25.9 (0.3)	0.22 (0.03)	21.2 (0.2)	0.03 (0.01)	23.0 (0.3)	0.16 (0.02)	25.7 (0.2)	0.13 (0.01)
12	20.1 (0.2)	0.08 (0.01)	23.4 (0.2)	0.10 (0.01)	24.5 (0.2)	0.11 (0.01)	22.3 (0.2)	0.16 (0.01)
13–15	20.3 (0.3)	0.07 (0.01)	23.6 (0.5)	0.09 (0.01)	23.7 (0.2)	0.16 (0.02)	25.3 (0.2)	0.16 (0.00)
16+	22.2 (0.2)	0.12 (0.01)	21.5 (0.2)	0.07 (0.01)	23.4 (0.3)	0.04 (0.01)	29.0 (0.3)	0.12 (0.04)
Parental race/ethnicity								
NH White^5^	21.0 (0.1)	0.12 (0.02)	21.8 (0.2)	0.07 (0.00)	23.2 (0.1)	0.12 (0.00)	24.8 (0.1)	0.15 (0.01)
NH Black	19.3 (0.4)	0.09 (0.02)	22.1 (0.8)	0.25 (0.07)	25.6 (0.2)	0.11 (0.01)	27.9 (0.3)	0.24 (0.01)
Hispanic	26.9 (0.3)	0.19 (0.02)	26.0 (0.3)	0.03 (0.03)	24.8 (0.1)	0.15 (0.00)	25.7 (0.2)	0.10 (0.01)
Other	17.8 (0.7)	−0.01 (0.02)	23.5 (1.2)	0.10 (0.02)	21.8 (0.8)	0.15 (0.18)	25.6 (1.1)	0.15 (0.02)
Annual household income								
Low	23.0 (0.2)	0.16 (0.06)	23.1 (0.2)	0.18 (0.02)	24.3 (0.1)	0.11 (0.00)	25.8 (0.1)	0.21 (0.01)
Middle	21.2 (0.1)	0.12 (0.02)	20.8 (0.2)	0.04 (0.01)	22.7 (0.2)	0.17 (0.02)	24.1 (0.1)	0.18 (0.01)
High	21.1 (0.2)	0.09 (0.01)	23.9 (0.3)	0.07 (0.01)	24.2 (0.2)	0.08 (0.01)	26.6 (0.3)	0.06 (0.05)
Parental marital status								
Other	19.2 (0.9)	0.11 (0.03)	25.6 (1.3)	0.06 (0.04)	23.2 (0.2)	0.11 (0.00)	23.6 (0.2)	0.19 (0.00)
Married	21.9 (0.1)	0.13 (0.01)	22.3 (0.1)	0.10 (0.00)	23.9 (0.1)	0.15 (0.01)	26.1 (0.1)	0.15 (0.00)
Parental MVPA/week^5^								
<3 times	22.3 (0.1)	0.13 (0.02)	21.3 (0.2)	0.15 (0.00)	21.9 (0.1)	0.06 (0.00)	25.5 (0.1)	0.11 (0.02)
≥3 times	21.1 (0.1)	0.12 (0.01)	23.4 (0.2)	0.06 (0.00)	25.1 (0.1)	0.17 (0.01)	25.4 (0.1)	0.20 (0.01)

1 For children, age- and sex-specific quintiles of BMI were used. For parents, sex-specific BMI quintiles were used. Weighted kappa values can be interpreted as follows: κ<0.20 =  poor agreement, κ between 0.20–0.40 =  fair agreement, κ between 0.40–0.60 =  moderate agreement, κ between 0.60–0.80 =  good agreement, and κ≥0.80 =  very good agreement.

2 The expected percent of agreement is 20%.

3 SEM are reported in parentheses and were obtained through Fay's balanced repeated replication (BRR) method of estimation.

4 Weighted kappa coefficients were calculated using the Cicchetti-Allison weight matrix (see text). All point estimates have taken MEPS’ sampling design into account. ^5^ NH, non-Hispanic;

5 MVPA, moderate-to-vigorous physical activity.

Source: Medical Expenditure Panel Survey, 2006–2007.

### Association between Parental Obesity Status and the Odds of Child Obesity

Among children of dual parent families, the proportions of children with only father being obese, only mother being obese, and both parents being obese were 17.3%, 13.6%, and 14.7%, respectively. The odds of being obese for children in dyads in which only the father was obese was 2.1 times greater compared to children with non-obese parents (i.e., neither parent was obese) (see Table 4); the odds was 1.9 times greater in dyads in which only the mother was obese and 3.2 times greater in dyads in which both parents were obese. Subgroup analyses did not find significant variation in the effects of parental obesity on the odds of child obesity by child’s age, household income, or parental age, race/ethnicity, education level, and frequency of physical activity. When comparing children whose parents were both obese to those whose parents were neither obese, the OR of being obese increased from 2.5 (95% CI:1.7, 3.6) for the 6–9 year-old age group to 7.5 (95% CI:4.4, 12.8) for 15–17 year-old age group.

**Table 4:** Association between child’s obesity and parents’ obesity status, stratified by child and parental characteristics as indicated^1^.

	Only father was obese^2^	Only mother was obese	Both father andmother were obese
	OR (95% CI)	OR (95% CI)	OR (95% CI)
Whole sample	2.1 (1.6, 2.8)^3^	1.9 (1.5, 2.4)	3.2 (2.5, 4.2)
Stratified by child sex			
Boys	2.4 (1.7, 3.4)	1.7 (1.3, 2.3)	3.3 (2.3, 4.6)
Girls	1.7 (1.2, 2.5)	2.0 (1.4, 2.9)	3.2 (2.3, 4.6)
By child age (years)			
6–9	1.6 (1.1, 2.3)	1.4 (1.0, 2.4)	2.5 (1.7, 3.6)
10–14	2.2 (1.3, 3.6)	2.1 (1.4, 3.2)	3.0 (2.1, 4.5)
15–17	3.7 (2.1, 6.6)	2.9 (1.6, 5.1)	7.5 (4.4, 12.8)
By annual household income			
Low income	1.9 (1.3, 2.9)	1.7 (1.2, 2.4)	2.5 (1.7, 3.5)
Middle income	2.3 (1.5, 3.6)	2.5 (1.7, 3.7)	4.2 (2.7, 6.5)
High income	2.1 (1.3, 3.3)	1.2 (0.6, 2.1)	3.5 (2.0, 5.9)
By paternal age (years)			
20–34	1.4 (0.8, 2.5)	2.4 (1.4, 4.3)	2.3 (1.4, 3.7)
35–49	2.5 (1.8, 3.4)	1.7 (1.2, 2.3)	3.3 (2.4, 4.6)
50–65	1.8 (0.9, 3.5)	2.1 (1.0, 4.2)	3.9 (1.9, 8.1)
By paternal race/ethnicity			
NH white^4^	2.3 (1.6, 3.2)	2.1 (1.5, 3.0)	3.2 (2.2, 4.7)
NH black	1.5 (0.7, 3.0)	1.9 (1.0, 3.4)	2.6 (1.3, 5.1)
Hispanic	2.5 (1.6, 3.8)	1.8 (1.2, 2.6)	4.6 (3.0, 7.0)
Other	2.4 (0.8, 7.4)	0.6 (0.1, 3.9)	3.8 (0.7, 21.9)
By paternal frequency of MVPA per week^5^			
<3 times	2.2 (1.6, 3.2)	1.8 (1.3, 2.5)	3.1 (2.2, 4.4)
≥3 times	2.0 (1.4, 3.0)	1.9 (1.4, 2.7)	3.3 (2.3, 4.8)
By paternal education (years)			
≤11 years (high school)	2.6 (1.6, 4.1)	1.5 (1.0, 2.2)	3.2 (2.0, 5.1)
12 years (high school)	2.0 (1.3, 3.1)	1.8 (1.2, 2.7)	2.8 (1.9, 4.2)
13–15 years (some college)	2.1 (1.1, 3.7)	2.5 (1.4, 4.5)	3.8 (2.0, 7.4)
≥16 years (≥college)	2.0 (1.1, 3.7)	1.9 (1.0, 3.8)	3.2 (1.7, 5.7)
By maternal age (years)			
20–34	2.0 (1.2, 3.4)	3.1 (2.0, 4.8)	2.8 (1.8, 4.3)
35–49	2.1 (1.5, 2.8)	1.5 (1.1, 2.0)	3.3 (2.4, 4.5)
50–65	3.6 (1.4, 9.4)	1.5 (0.4, 5.8)	4.1 (1.2, 13.6)
By maternal race/ethnicity			
NH white	2.1 (1.5, 3.0)	2.1 (1.5, 2.9)	3.3 (2.3, 4.7)
NH black	1.4 (0.7, 3.0)	1.7 (0.9, 3.2)	2.6 (1.4, 5.2)
Hispanic	2.9 (1.7, 4.9)	1.9 (1.3, 2.8)	4.2 (2.7, 6.7)
Other	5.0 (1.3, 19.0)	0.7 (0.1, 4.0)	3.8 (0.8, 19.5)
By maternal frequency of MVPA per week			
<3 times	2.4 (1.6, 3.5)	2.4 (1.7, 3.4)	3.2 (2.2, 4.6)
≥3 times	1.8 (1.3, 2.6)	1.5 (1.1, 2.1)	3.1 (2.2, 4.4)
By maternal education (years)			
≤11 years (high school)	3.0 (1.9, 4.9)	2.6 (1.7, 3.9)	4.0 (2.6, 6.1)
12 years (high school)	1.6 (1.0, 2.8)	1.8 (1.2, 2.6)	2.8 (1.9, 4.4)
13–15 years (some college)	1.3 (0.7, 2.2)	1.7 (0.9, 2.9)	3.4 (2.1, 5.6)
≥16 years (≥college)	3.5 (2.1, 6.0)	1.5 (0.7, 3.1)	3.2 (1.5, 6.6)

1 Results are reported based on logistic regression models adjusted for child sex and age, household income, and parental age, education, race/ethnicity, and physical activity. The reference group was children whose parents were neither obese.

2 Obese was defined as BMI ≥30 kg/m^2^.

3 The odds ratio (OR)for child obesity was 2.1, comparing children for whom only their father was obese to those whose father and mother were both not obese.

4 NH, non-Hispanic; ^5^ MVPA, moderate-to-vigorous physical activity.

Source: Medical Expenditure Panel Survey, 2006–2007.

### Predictors of Patterns of Parent-child Agreement in Overweight Status

Child’s sex and age, household income, and parental education, race/ethnicity, and frequency of physical activity were all associated with patterns of parent-child agreement in weight status (see Table 5). Compared to girls, boys were more likely to be part of (1) the dyads composed of a normal weight mother and an overweight child (OR = 1.9 [95% CI: 1.5, 2.3]) or (2) the dyads in which both mother and child were overweight (OR = 1.3 [95% CI: 1.1, 1.5]) than (3) the dyads where both mother and child were normal weight. Compared to mother-child pairs with a 6–9 year-old child, the pairs with children ≥10 years old were less likely to be in (1) the dyads in which both the mother and child were overweight or (2) the dyads in which the mother was of normal weight and the child overweight than (3) the dyads in which neither the mother nor the child were overweight.

**Table 5:** The associations between socio-demo-economic characteristics and patterns of concordance between parent-child body weight status: Multinomial logistic models^1^.

	Model 1: Mother-child dyads (Compared tonormal weight mother and child)			Model 2: Father-child dyads (Compared tonormal weight father and child)			
Characteristic (vs. reference group)	Overweight motherand child^2^	Overweight mother, normal weight child	Normal weight mother, overweight child		Overweight fatherand child	Overweight father, normal weight child	Normal weight father, overweight child
	OR (95% CI)	OR (95% CI)	OR (95% CI)		OR (95% CI)	OR (95% CI)	OR (95% CI)
**Boy (vs. girl)**	1.3 (1.1, 1.5)	1.0 (0.9, 1.2)	1.9 (1.5, 2.3)		1.6 (1.3, 1.9)	1.1 (1.0, 1.3)	1.4 (1.2, 1.7)
**Child age (vs. 6–9 years)**							
10–14	0.7 (0.6, 0.9)	1.0 (0.8, 1.2)	0.6 (0.4, 0.7)		0.6 (0.5, 0.8)	0.9 (0.7, 1.1)	0.6 (0.5, 0.7)
15–17	0.5 (0.4, 0.7)	1.0 (0.8, 1.3)	0.4 (0.3, 0.5)		0.5 (0.4, 0.7)	1.0 (0.7, 1.3)	0.4 (0.3, 0.5)
**Annual household income (vs. high income)**							
Low	2.1 (1.6, 2.9)	1.5 (1.1, 1.9)	1.4 (1.0, 1.9)		1.3 (0.9, 1.9)	1.0 (0.8, 1.4)	2.4 (1.8, 3.3)
Middle	1.8 (1.4, 2.4)	1.5 (1.1, 1.8)	1.5 (1.1, 1.9)		1.4 (1.0, 1.9)	1.1 (0.9, 1.4)	1.8 (1.3, 2.5)
**Parental age (vs. 31–49 years)**							
20–30 years	0.9 (0.7, 1.2)	0.8 (0.7, 1.1)	0.8 (0.6, 1.0)		0.7 (0.5, 1.0)	0.6 (0.4, 0.9)	0.7 (0.4, 1.1)
50–65 years	1.0 (0.7, 1.5)	1.0 (0.7, 1.3)	0.7 (0.4, 1.2)		0.7 (0.5, 1.0)	0.7 (0.5, 1.0)	0.7 (0.4, 1.1)
**Parental race/ethnicity (vs. NH white)** 3							
NH black	3.3 (2.4, 4.6)	2.5 (1.9, 3.2)	1.5 (1.1, 2.1)		1.7 (1.1, 2.5)	0.9 (0.6, 1.4)	2.2 (1.4, 2.5)
Hispanic	1.9 (1.4, 2.4)	1.4 (1.1, 1.9)	1.4 (1.1, 1.9)		1.9 (1.3, 2.6)	1.0 (0.7, 1.3)	1.0 (0.6, 1.6)
Other	0.7 (0.4, 1.1)	0.5 (0.3, 0.8)	1.3 (0.9, 1.9)		0.4 (0.2, 0.7)	0.3 (0.2, 0.5)	1.6 (1.0, 2.7)
**Parental education (vs. 16 years)**							
≤11 years	3.0 (2.1, 4.3)	1.9 (1.3, 2.8)	2.5 (1.7, 3.8)		2.1 (1.3, 3.3)	1.1 (0.8, 1.7)	2.3 (1.3, 4.0)
12 years	2.7 (2.0, 3.6)	1.8 (1.3, 2.4)	1.8 (1.3, 2.6)		2.1 (1.4, 3.0)	1.2 (0.9, 1.8)	1.9 (1.2, 3.1)
13–15 years	2.1 (1.5, 2.9)	1.6 (1.2, 2.1)	1.5 (1.1, 2.0)		1.7 (1.1, 2.6)	1.3 (0.9, 1.9)	1.5 (0.9, 2.5)
**Parental marriage status**							
Other vs. married	1.0 (0.8, 1.3)	1.0 (0.8, 1.3)	1.2 (0.9, 1.5)		1.0 (0.6, 1.5)	0.8 (0.5, 1.2)	1.1 (0.7, 1.7)
**Parental frequency of MVPA per week** 4							
≥3 vs. <3 times	0.5 (0.4, 0.6)	0.5 (0.4, 0.7)	0.9 (0.8, 1.2)		0.7 (0.6, 0.9)	0.8 (0.6, 1.0)	0.9 (0.6, 1.2)

1 The odds ratio (OR) for specific patterns of concordance in dyad overweight status as observed across socio-demographic and socioeconomic characteristics. All the variables shown in this table (Table 5) were specified in the multinomial logistic model. For each model for the father-child dyads, the reference group for the dependent variable were non-overweight father-non-overweight child dyads. The same held for mother-child dyads; the reference group for the dependent variable for each model were non-overweight mother-non-overweight child dyads.

2 Overweight status was defined as BMI ≥25 kg/m^2^ for adults and BMI ≥85^th^ percentile for children.

3 NH, non-Hispanic.

4 MVPA, moderate-to-vigorous physical activity.

Source: Medical Expenditure Panel Survey, 2006–2007.

Lower household income was associated with increased risks of either mother or the child being overweight in the mother-child dyads. For father-child dyads, lower household income was only associated with the child’s overweight but not the father’s. Compared with the pairs in which either parent was NH white, those pairs in which either parent was of NH black or Hispanic race/ethnicity were at increased risk of being in mother-child pairs with either an overweight child or overweight mother, or in father-child pairs with an overweight child. Children whose parents had less education were more likely to be in mother-child pairs that included at least one overweight party, father-child pairs in which both father and child were overweight, or father-child pairs in which only the child was overweight. Dyads with a physically active parent were less likely to have overweight parent(s) and children. Parental age and marital status were not associated with patterns of parent-child agreement.

## Discussion

We examined parent-child resemblance in body weight status and its association with other socio-demographic and socioeconomic factors in the US using nationally representative data collected as part of the 2006 and 2007 MEPS. Overall, we observed a weak parent-child resemblance and noticed some differences across population subgroups. For example, Pearson correlations between parent and child BMI levels were low; most were <0.20 for the various parent-child dyad types and socio-demographic subgroups. Other measures, such as the weighted kappa coefficients and percent agreements between parent and children’s BMI quintiles, were also low. Child’s sex and age, family income, and parental education, race/ethnicity, and moderate-to-vigorous physical activity affected parent-child agreement in overweight status.

The degree of parent-child resemblance in body weight status we observed is weaker than that reported by some studies, but it is generally consistent with other research. For example, a small US study among 101 healthy pre-pubertal girls and their biological parents reported stronger correlation coefficients for BMI in mother-daughter pairs (r = 0.34) and father-daughter pairs (r = 0.44) [28]. A multi-country study conducted in Germany, Austria and Switzerland also reported a strong mother-child correlation for BMI, which was greater than correlation in father-child dyads (r  = 0.29 vs. 0.18, respectively, p<0.001) [29]. One large study of more than 25,000 twin pairs and 50,000 of their biological or adoptive family members reported a mean correlation coefficient of 0.19 for BMI in biological parent-offspring pairs, which was similar to our findings [30]. Conversely, another US study involving 76 girls aged 9–13 years and their mothers did not report a significant mother-daughter resemblance in BMI [31].

Previous studies and ours also showed mixed findings on the gender-differences in parent-child resemblance. For example, our study showed little difference between mother-daughter dyads and the other dyad types (e.g., mother-son, father-daughter, and father-son). These distinctions could result from the use of different study samples (e.g., nationally representative versus specific, small, and non-representative), different modalities of measurements (e.g., self-reported weight or height versus measured), and other social-environmental factors that vary across populations (e.g., those related to school meals and physical activity, which may in turn affect children's weight status). In addition, our study included both biological and non-biological parents, while the above-cited US studies either included biological parents only or can separate biological and adoptive parents.

In our study, we also compared the parent-child resemblance across socioeconomic groups. We found some differences by race/ethnicity; agreement between parent-child BMI quintiles was greater in dyads in which the father was Hispanic or the mother was NH black. After controlling for other socioeconomic factors, these correlations between parent-child BMI did not significantly differ across racial/ethnic or household income groups. Nevertheless, certain SES factors were associated with the variation in parent-child resemblance in weight status. For example, lower parental education was associated with stronger resemblance in BMI in both father-son and mother-son dyads. In general, those factors were also related to childhood obesity risk. Identification of these shared factors can help researchers better understand the parental influences of childhood obesity.

MEPS data did not differentiate biological and adoptive parents, so we cannot determine how much of the parent-child resemblance in weight status was attributable to genetic versus non-genetic factors. Studies distinguishing between adopted and biological children suggest that genetic factors play a more important role in parent-child resemblance in weight status than environmental factors [28], [30]. For example, in the large US study discussed above that included both biological and adopted children and their family members, the mean correlation coefficient between the BMI of biological parent-offspring pairs was stronger than that for non-biological parent-offspring pairs (0.19 vs. 0.06, respectively) [30].

Previous research regarding the relatively weak parent-children resemblance in energy balance-related behaviors may help explain the relatively weak resemblance in weight status. In a previous study based on nationally representative data for the US, the parent-child correlation in overall quality of dietary intake was 0.26. Correlations ranged from 0.20 to 0.33 for most other individual dietary intake measures, such as for nutrients and food groups [32]. Another US study found weak parent-child correlations in physical activity, where r ranged from 0.03 in mother-son pairs to 0.18 in father-son pairs [33]. These weak resemblances in parent-child dietary intakes and physical activity indicate that broader social, environmental and behavioral forces (e.g., factors beyond parental eating and physical activity behaviors) likely affect children’s weight status, so contributing to the relatively weak parent-child resemblance in weight status. However, it is also possible that resemblances in eating and physical activity behaviors may be underestimated due to measurement errors.

Our study found that US children living with two obese parents were 3.2 times more likely to be obese than children living with non-obese parents. One previous US study reported that the odds of being obese at age 21–29 years were more than two times greater if both parents were obese than if only one parent was obese [14]. Some studies from other countries have reported similar findings. According to a study in China, the OR of being obese was 28.5 (95% CI: 15.1, 53.7) for girls for whom both parents were obese compared to those with normal-weight parents [34].

The present study has several important strengths, including: the use of data from a large nationally representative recent dataset, the application of appropriate statistical methods, and the thorough assessment of parent-children resemblance in weight status and its correlates based on a varied set of analytic approaches and measures. Correlation coefficients capture the degree of dependency between two continuous variables through the full range of observations, though it can be subject to measurement error at the tail ends of distributions. Kappa coefficients, on the other hand, could be less sensitive to measurement error, but this method is only applied to categorical data. Besides, while it is certainly possible to convert continuous data to categories (as we did here), the categorization can blunt the richer, inherent detail within continuous measurements. Although OR handles binary outcomes only, using logistic regression can estimate OR that reveals the independent association between predictors and the binary outcome while controlling for confounders. Percent agreements reflect the crude joint distribution of two categorical or categorized variables through the values in the diagonal cells; these serve as a crude version of the kappa coefficient before expected distributions are taken into account. Since these measures of resemblance have their own strength and limitation, we explored the resemblance using all the methods to explore the parent-child resemblance in weight status from different perspectives.

One major limitation of our study is the use of self-reported weight and height. This may result in some information bias, although previous studies have documented the accuracy and reliability of self-reported weight and height for different populations [35], [36], [37], [38], [39]. Another limitation is that BMI was not available or missing for 21% of the children aged 6–17 years in the MEPS 2006 and 2007 data files. About 22% children were not included for final analysis because their father had missing value of BMI; about 23% children were not included for analysis because their mother had missing value of BMI. This may have led to selection bias and affected the representativeness of our results. However, the only significant difference we observed between the children included in our analyses and the children who were excluded (i.e., had missing or extreme BMI values of children or of parents) was that the latter group was on average 1.7–1.8 years older. Furthermore, MEPS contains limited information on diet and physical activity. Thus, it was not possible to fully test the effects of these factors on parent-child resemblance in weight status.

In conclusion, parent-child resemblance in BMI and weight status was weak in the US population. Some socio-demographic and socioeconomic factors such as child age, parental education and race/ethnicity, and household income exerted influence on the degree of resemblance. Children with two obese parents were more than three times as likely to be obese than children with two non-obese parents. Different measures of resemblance can yield different conclusions. Future research is needed to help better understand the factors that affect parent-child resemblance in body weight status and guide childhood obesity prevention efforts.
